# *Gardnerella* Species and Their Association With Bacterial Vaginosis

**DOI:** 10.1093/infdis/jiae026

**Published:** 2024-01-24

**Authors:** Matthew M Munch, Susan M Strenk, Sujatha Srinivasan, Tina L Fiedler, Sean Proll, David N Fredricks

**Affiliations:** Vaccine and Infectious Disease Division, Fred Hutchinson Cancer Center, Seattle, Washington, USA; Vaccine and Infectious Disease Division, Fred Hutchinson Cancer Center, Seattle, Washington, USA; Vaccine and Infectious Disease Division, Fred Hutchinson Cancer Center, Seattle, Washington, USA; Vaccine and Infectious Disease Division, Fred Hutchinson Cancer Center, Seattle, Washington, USA; Vaccine and Infectious Disease Division, Fred Hutchinson Cancer Center, Seattle, Washington, USA; Vaccine and Infectious Disease Division, Fred Hutchinson Cancer Center, Seattle, Washington, USA; Department of Medicine, University of Washington, Seattle, Washington, USA

**Keywords:** *Gardnerella*, bacterial vaginosis, *cpn60*, microbiome

## Abstract

**Background:**

Bacterial vaginosis (BV) is a condition marked by high vaginal bacterial diversity. *Gardnerella vaginalis* has been implicated in BV but is also detected in healthy women. The *Gardnerella* genus has been expanded to encompass 6 validly named species and several genomospecies. We hypothesized that particular *Gardnerella* species may be more associated with BV.

**Methods:**

Quantitative polymerase chain reaction (PCR) assays were developed targeting the *cpn60* gene of species groups including *G. vaginalis*, *G. piotii/pickettii, G. swidsinskii/greenwoodii,* and *G. leopoldii.* These assays were applied to vaginal swabs from individuals with (n = 101) and without BV (n = 150) attending a sexual health clinic in Seattle, Washington. Weekly swabs were collected from 42 participants for up to 12 weeks.

**Results:**

Concentrations and prevalence of each *Gardnerella* species group were significantly higher in participants with BV; 91.1% of BV-positive participants had 3 or more *Gardnerella* species groups detected compared to 32.0% of BV-negative participants (*P* < .0001). BV-negative participants with 3 or more species groups detected were more likely to develop BV within 100 days versus those with fewer (60.5% vs 3.7%, *P* < .0001).

**Conclusions:**

These results suggest that BV reflects a state of high *Gardnerella* species diversity. No *Gardnerella* species group was a specific marker for BV.

## Teaser

Prevalence and concentrations of 4 *Gardnerella* species groups as measured by quantitative PCR were significantly associated with bacterial vaginosis (BV). Detection of multiple *Gardnerella* species was a risk factor for BV development, and none were a specific marker for BV.

Bacterial vaginosis (BV) is the most common cause of vaginal discharge in reproductive age women worldwide and is marked by high bacterial diversity, increased abundance of BV-associated anaerobic bacteria, and decreased abundance of lactobacilli in the vagina [1–3]. BV has been associated with a wide range of negative health outcomes such as increased risk of preterm birth [4], acquisition of sexually transmitted infections [5, 6] and pelvic inflammatory disease [7]. BV etiology remains incompletely understood and the heterogeneous, polymicrobial nature of this condition has made efforts to study BV challenging. Hypothetical models have been advanced to help explain the pathogenesis of BV [8].

*Gardnerella* has been implicated in the development of BV since the 1950s and was the first bacterium linked to nonspecific vaginitis, now known as BV [9]. Previous studies have shown increased abundance of vaginal *Gardnerella* in women with BV [10, 11] and *Gardnerella* has been associated with adherent biofilms in BV [12]. There is evidence that *Gardnerella* can be sexually transmitted [13, 14], and it may be a keystone species in the development of BV [8, 14]. However, *Gardnerella* has also been detected by polymerase chain reaction (PCR) in more than 50% of women without BV, calling into question its role in BV etiology [15, 16]. Formerly classified as *Gardnerella vaginalis*, the *Gardnerella* genus has been shown to consist of at least 13 unique genomospecies, 6 of which have been validly named: *Gardnerella vaginalis*, *Gardnerella piotii*, *Gardnerella swidsinskii*, *Gardnerella leopoldii, Gardnerella pickettii, and Gardnerella greenwoodii* [17, 18]. Vaginal colonization with multiple *Gardnerella* species has been demonstrated in prior studies [10, 13, 19–24] and highlights the need to further understand the prevalence and role of each species within the *Gardnerella* genus. We hypothesized that a subset of *Gardnerella* species is more prevalent and abundant in women with BV, thereby contributing to the etiology of BV due to pathogenic characteristics of these species. In contrast, we expected women without BV to be colonized by other *Gardnerella* species, potentially explaining the high prevalence of *Gardnerella* commonly noted in women without BV.

Previous studies have often utilized the 16S ribosomal RNA (rRNA) gene for *Gardnerella* identification; however, all *Gardnerella* species are more than 98.5% identical in this region [17], necessitating the use of other phylogenetic marker genes to differentiate *Gardnerella* species. We developed 4 quantitative PCR (qPCR) assays targeting the *cpn60* gene of species groups including *G. vaginalis*, *G. piotii/pickettii*, *G. swidsinskii/greenwoodii,* and G. *leopoldii.* These assays were applied to vaginal swabs collected from 101 study participants with BV and 150 participants without BV. Longitudinal weekly swabs from a subset of 42 participants (16 BV positive and 26 BV negative) were used to monitor vaginal *Gardnerella* population dynamics over time and response to metronidazole treatment.

## METHODS

### Study Population and Sample Collection

From October 2012 to August 2015, vaginal swabs were collected from 251 nonpregnant study participants with (n = 101) and without (n = 150) BV attending the Public Health Seattle and King County Sexual Health Clinic. The overall study population included individuals assigned female sex at birth in Seattle, Washington, and individuals with and without risk factors for BV to include previous history of BV, identifying as black and/or Hispanic, or high number of sexual partners [25]. The study was approved by the Fred Hutchinson Cancer Center institutional review board (protocol No. 7683) and all study participants provided informed consent. Vaginal swabs were collected by clinicians at baseline and subsequent follow-up clinic visits. Participants were considered BV positive when at least 3 of 4 Amsel clinical criteria were met (symptomatic BV) [26]. Vaginal smears of all clinic samples were prepared on glass slides for Gram stain and assessment of BV by Nugent scoring [27]. Participants with symptomatic BV were treated with metronidazole at each clinic visit following Centers for Disease Control and Prevention guidelines. Most study participants did not remain enrolled beyond 100 days, and this was used as a cutoff for analysis of BV development. A subset of 42 participants provided at least 10 additional weekly self-collected vaginal swabs for up to 12 weeks from baseline and these samples were used for longitudinal analysis. Behavioral data were collected via questionnaires at clinic visits and via daily diaries. A more detailed description of the study population and sample collection can be found in the Supplementary Methods.

### DNA Extraction and qPCR

DNA was extracted from vaginal swabs as described in Supplementary Methods. Total *Gardnerella* concentrations were quantified using a previously developed qPCR assay targeting the *Gardnerella* 16S rRNA gene [28]. Using the species designations proposed by Vaneechoutte et al [17] and Sousa et al [18], 4 qPCR assays were developed targeting the *cpn60* gene of the following species groups: *G. vaginalis* and *Gardnerella* genomospecies 2, *G. piotii* and *G. pickettii*, *G. leopoldii*, *G. swidsinskii,* and *G. greenwoodii* (*Gardnerella* genomospecies sp 9 and 10 also detected with reduced sensitivity, see Supplementary Table 1). We report the species groups detected by these assays here as *G. vaginalis*, *G. piotii/pickettii*, *G. swidsinskii/greenwoodii*, and *G. leopoldii*. Assay conditions and oligonucleotide sequences are shown in Supplementary Table 1 and further described in the Supplementary Methods.

### Statistical Analysis

Figures were created using matplotlib, seaborn, and statannotations Python packages [29–31]. Statistical analysis was performed using GraphPad Prism 9.5.1 for Windows and the psych R Statistical software package [32, 33]. Significance of qPCR data was calculated using the Mann-Whitney test. Relative risk confidence intervals (CIs) were calculated using Koopman asymptotic score and significance was calculated using Fisher exact test. Heatmap correlations were calculated using Spearman correlation.

## RESULTS

### Study Population

The 251 participants enrolled in the study ranged from 19 to 49 years of age; 51.8% identified as white, 35.4% as black, and 10.4% as other; 7.2% of participants identified as Hispanic. Of the participants, 8.8% did not identify as black or Hispanic and reported both no history of BV and 10 or fewer lifetime sex partners (no BV risk factors). In total, 101 participants were BV positive at baseline by Amsel criteria and 150 were BV negative; 115 participants had Nugent scores indicative of no BV (0–3), 32 had Nugent scores indicative of intermediate microbiota (4–6), and 103 had Nugent scores indicative of BV (7–10). Thirty-nine participants were Amsel negative but had Nugent scores >3 (asymptomatic BV/intermediate microbiota); 80 of 101 (79.2%) participants with symptomatic BV reported a history of BV compared to 86 of 150 (57.3%) of BV-negative participants (Table 1). Additional participant characteristics are shown in Supplementary Table 2.

**Table 1.** Participant Characteristics at Baseline

Characteristic	All Participants	BV Negative, Amsel	BV Positive, Amsel	Longitudinal Participants	Longitudinal BV Negative, Amsel	Longitudinal BV Positive, Amsel
No.	251	150	101	42	26	16
Age, y, median ± SD, range	30 ± 8.6, 19–49	30 ± 8.9, 19–49	30 ± 8.1, 19–49	27 ± 7.6, 19–49	27 ± 8.2, 19–49	29 ± 6.7, 22–44
Age group, y						
19–25	63 (25.1)	37 (24.7)	26 (25.7)	14 (33.3)	10 (38.5)	4 (25.0)
26–35	105 (41.8)	61 (40.6)	44 (43.6)	21 (50.0)	12 (46.1)	9 (56.3)
36–49	78 (31.1)	51 (34.0)	27 (26.7)	6 (14.3)	4 (15.4)	2 (12.5)
No response	5 (2.0)	1 (0.7)	4 (4.0)	1 (2.4)	0 (0)	1 (6.2)
Race						
White	130 (51.8)	86 (57.3)	44 (43.6)	20 (47.6)	13 (50.0)	7 (43.8)
Black	89 (35.4)	40 (26.7)	49 (48.5)	17 (40.5)	9 (34.6)	8 (50.0)
Other	26 (10.4)	20 (13.3)	6 (5.9)	5 (11.9)	4 (15.4)	1 (6.2)
No response	6 (2.4)	4 (2.7)	2 (2.0)	0 (0)	0 (0)	0 (0)
Ethnicity						
Hispanic	18 (7.2)	11 (7.3)	7 (6.9)	2 (4.8)	2 (7.7)	0 (0)
Non-Hispanic	200 (79.7)	122 (81.4)	78 (77.2)	35 (83.3)	21 (80.8)	14 (87.5)
No response	33 (13.1)	17 (11.3)	16 (15.9)	5 (11.9)	3 (11.5)	2 (12.5)
Nugent score						
0–3	115 (45.8)	110 (73.3)	5 (5.0)	17 (40.5)	16 (61.5)	1 (6.2)
4–6	32 (12.8)	16 (10.7)	16 (15.8)	2 (4.8)	2 (7.7)	0 (0)
7–10	103 (41.0)	23 (15.3)	80 (79.2)	23 (54.7)	8 (30.8)	15 (93.8)
No Nugent score	1 (0.4)	1 (0.7)	0 (0)	0 (0)	0 (0)	0 (0)
Reported History of BV						
Yes	166 (66.1)	86 (57.3)	80 (79.2)	31 (73.8)	17 (65.4)	14 (87.5)
No	79 (31.5)	61 (40.7)	18 (17.8)	10 (23.8)	8 (30.8)	2 (12.5)
Do not know or no response	6 (2.4)	3 (2.0)	3 (3.0)	1 (2.4)	1 (3.8)	0 (0)
BV Development within 100 d						
1 or more follow-up visits	213 (84.9)	124 (82.7)	89 (88.1)	42 (100)	26 (100)	16 (100)
Symptomatic BV at any follow-up	75 (29.9)	29 (23.4)	46 (51.7)	26 (61.9)	12 (46.2)	14 (87.5)

Data are No. (%) except where indicated.

Abbreviation: BV, bacterial vaginosis.

### Prevalence and Concentrations of *Gardnerella* Species

**Figure 1.**
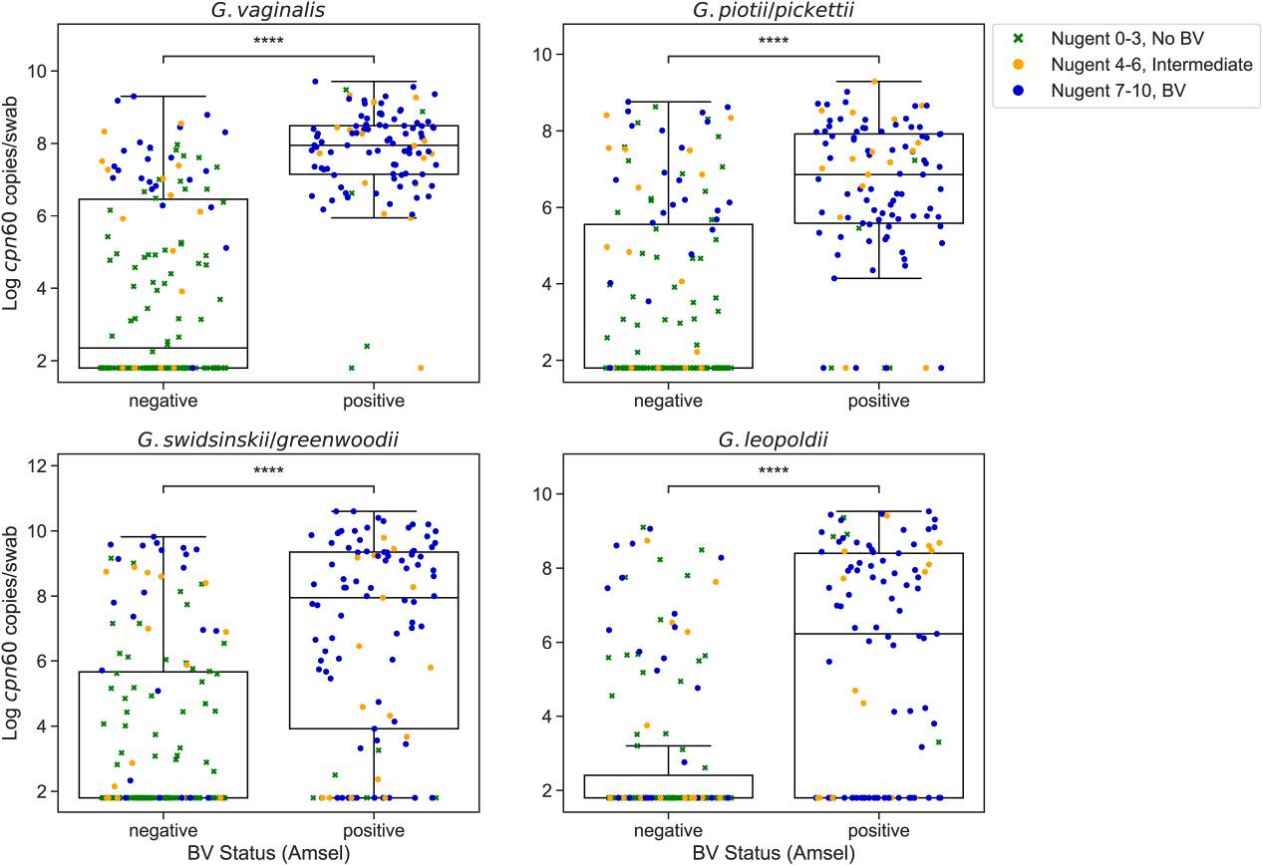
*Gardnerella cpn60* gene concentrations in participants with and without symptomatic BV. Log transformed baseline *Gardnerella cpn60* gene concentrations as measured by qPCR in BV-negative (n = 150) and BV-positive (n = 101) participants by Amsel criteria. Each datapoint represents a participant at baseline and is colored and shaped according to BV diagnosis by Nugent score. Boxes show lower quartile, median, and upper quartile with whiskers extended to 1.5 times the interquartile range. Median *Gardnerella* species group *cpn60* concentrations were significantly higher in BV-positive participants. ****P < .0001, Mann-Whitney test. Abbreviations: BV, bacterial vaginosis; qPCR, quantitative polymerase chain reaction.

**Figure 2.**
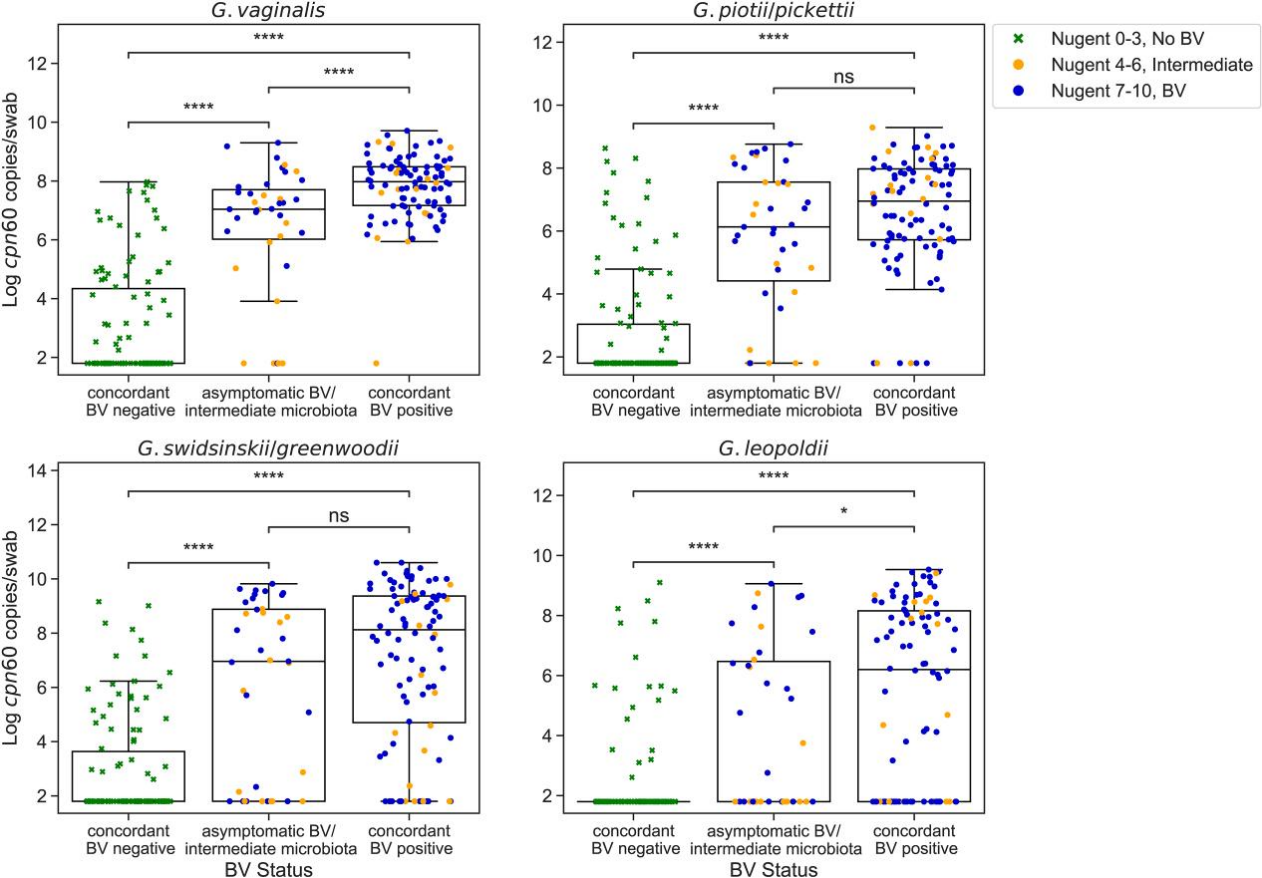
*Gardnerella cpn60* concentrations in participants with Amsel and Nugent score concordant and discordant BV diagnoses. Log transformed baseline *Gardnerella cpn60* gene concentrations in participants with concordant BV-negative diagnosis (Amsel negative, Nugent score 0–3, n = 110), asymptomatic BV/intermediate microbiota (Amsel negative, Nugent score 4–10, n = 39), and concordant BV-positive diagnosis (Amsel positive, Nugent score 4–10, n = 96). Each datapoint represents a participant at baseline and is colored and shaped according to BV diagnosis by Nugent score. Boxes show lower quartile, median, and upper quartile with whiskers extended to 1.5 times the interquartile range. Median *Gardnerella* species group *cpn60* concentrations were significantly higher in participants with concordant BV and asymptomatic BV/intermediate microbiota compared to concordant BV-negative participants. *P < .05, ****P < .0001, Mann-Whitney test. Abbreviations: BV, bacterial vaginosis; ns, not significant.

*Gardnerella* was detected via 16S rRNA gene qPCR in 100 of 101 (99.0%) Amsel BV-positive participants and 112 of 150 (74.7%) Amsel-negative participants. Using the qPCR assays developed in this study targeting the *cpn60* gene, *G. vaginalis* was most prevalent (69.7%), followed by *G. piotii/pickettii* (62.9%), *G. swidsinskii/greenwoodii* (58.6%), and *G. leopoldii* (41.0%). All 4 species groups were more frequently detected in BV-positive participants compared to BV-negative participants at baseline as measured by both Amsel criteria and Nugent score (Table 2 and Supplementary Table 3). Median concentrations of each *Gardnerella* species group were significantly higher in Amsel BV-positive participants compared to Amsel-negative participants (*P* < .0001; Figure 1), and significantly higher in participants with Nugent scores of 4–6 (intermediate microbiota) or 7–10 (BV) compared to those with scores of 0–3 (no BV) (Supplementary Figure 1). Study participants with concordant BV (Amsel positive, Nugent 7–10, n = 96), as well as participants with asymptomatic BV/intermediate microbiota (Amsel negative, Nugent 4–10, n = 39), had significantly higher concentrations of all 4 *Gardnerella* species groups compared to concordant BV-negative participants (Amsel negative, Nugent 0–3, n = 110; *P* < .0001; Figure 2). Median total bacterial concentration was significantly higher in participants with symptomatic BV as measured by a broad-range 16S rRNA gene qPCR assay (8.65 vs 8.02 log_10_ gene copies/swab, *P* < .0001).

**Table 2.** Performance Characteristics of *Gardnerella cpn60* qPCR Assays for BV Detection Based on Amsel Criteria at Baseline

qPCR Assay Detection	Total, No. (%) (n = 251)	BV Positive, No. (%) (n = 101)	BV Negative, No. (%) (n = 150)	Sensitivity, %	Specificity, %	PPV, %	NPV, %	RR	95% CI	*P* Value
Bacteria present above assay threshold										
*Gardnerella* 16S rRNA	212 (84.5)	100 (99.0)	112 (74.7)	99.0	25.3	47.2	97.4	18.4	3.6–104.3	<.0001
*G. vaginalis*	175 (69.7)	99 (98.0)	76 (50.7)	98.0	49.3	56.6	97.4	21.5	6.2–78.5	<.0001
*G. piotii*/*pickettii*	158 (62.9)	92 (91.1)	66 (44.0)	91.1	56.0	58.2	90.3	6.0	3.3–11.4	<.0001
*G. swidsinskii*/*greenwoodii*	147 (58.6)	83 (82.2)	64 (42.7)	82.2	57.3	56.5	82.7	3.3	2.1–5.1	<.0001
*G. leopoldii*	103 (41.0)	65 (64.4)	38 (25.3)	64.4	74.7	63.1	75.7	2.6	1.9–3.6	<.0001
3 or more *Gardnerella cpn60* species groups	140 (55.8)	92 (91.1)	48 (32.0)	91.1	68.0	65.7	91.9	8.1	4.4–15.4	<.0001
Quantity detected above median concentration of positive samples										
*Gardnerella* 16S rRNA, >1.70e8 copies/swab	106 (42.2)	82 (81.2)	24 (16.0)	81.2	84.0	77.4	86.9	5.9	3.9–9.1	<.0001
*G. vaginalis,* >2.45e7 copies/swab	87 (34.7)	69 (68.3)	18 (12.0)	68.3	88.0	79.3	80.5	4.1	3.0–5.7	<.0001
*G. piotii*/*pickettii,* >3.48e6 copies/swab	79 (31.5)	54 (53.5)	25 (16.7)	53.5	83.3	68.4	72.7	2.5	1.9–3.3	<.0001
*G. swidsinskii*/*greenwoodii,* >6.25e7 copies/swab	73 (29.1)	53 (52.5)	20 (13.3)	52.5	86.7	72.6	73.0	2.7	2.0–3.6	<.0001
*G. leopoldii,* >4.28e7 copies/swab	51 (20.3)	40 (39.6)	11 (7.3)	39.6	92.0	76.9	69.4	2.5	1.9–3.2	<.0001

Abbreviations: BV, bacterial vaginosis; CI, confidence interval; NPV, negative predictive value; PPV, positive predictive value; qPCR, quantitative polymerase chain reaction; RR, relative risk; rRNA, ribosomal RNA.

### *Gardnerella* Species: Sensitivity and Specificity for BV

*Gardnerella* 16S rRNA gene detection at baseline had 99.0% sensitivity, 25.3% specificity, and a risk ratio (RR) of 18.4 (95% CI, 3.6–104.3; *P* < .001) for symptomatic BV. *Gardnerella* species group detection using *cpn60* assays at baseline had the following sensitivities, specificities, and RR for symptomatic BV, respectively: *G. vaginalis* (98.0%, 49.3%, 21.5, *P* < .001), *G. piotii/pickettii* (91.1%, 56.0%, 6.0, *P* < .001), *G. swidsinskii/greenwoodii* (82.2%, 57.3%, 3.3, *P* < .001), and *G. leopoldii* (64.4%, 74.7%, 2.6, *P* < .001). Detection of 3 or more *Gardnerella* species groups at baseline had 91.1% sensitivity, 68.0% specificity, and a RR of 8.1 (95% CI, 4.4–15.4; *P* < .001) for symptomatic BV. Detection above the median concentration of positive samples for each *Gardnerella* species group improved specificity for symptomatic BV but decreased sensitivity (Table 2). Similar results were found when using Nugent score for BV diagnosis (Supplementary Table 4).

### Cooccurrence of *Gardnerella* species

**Figure 3.**
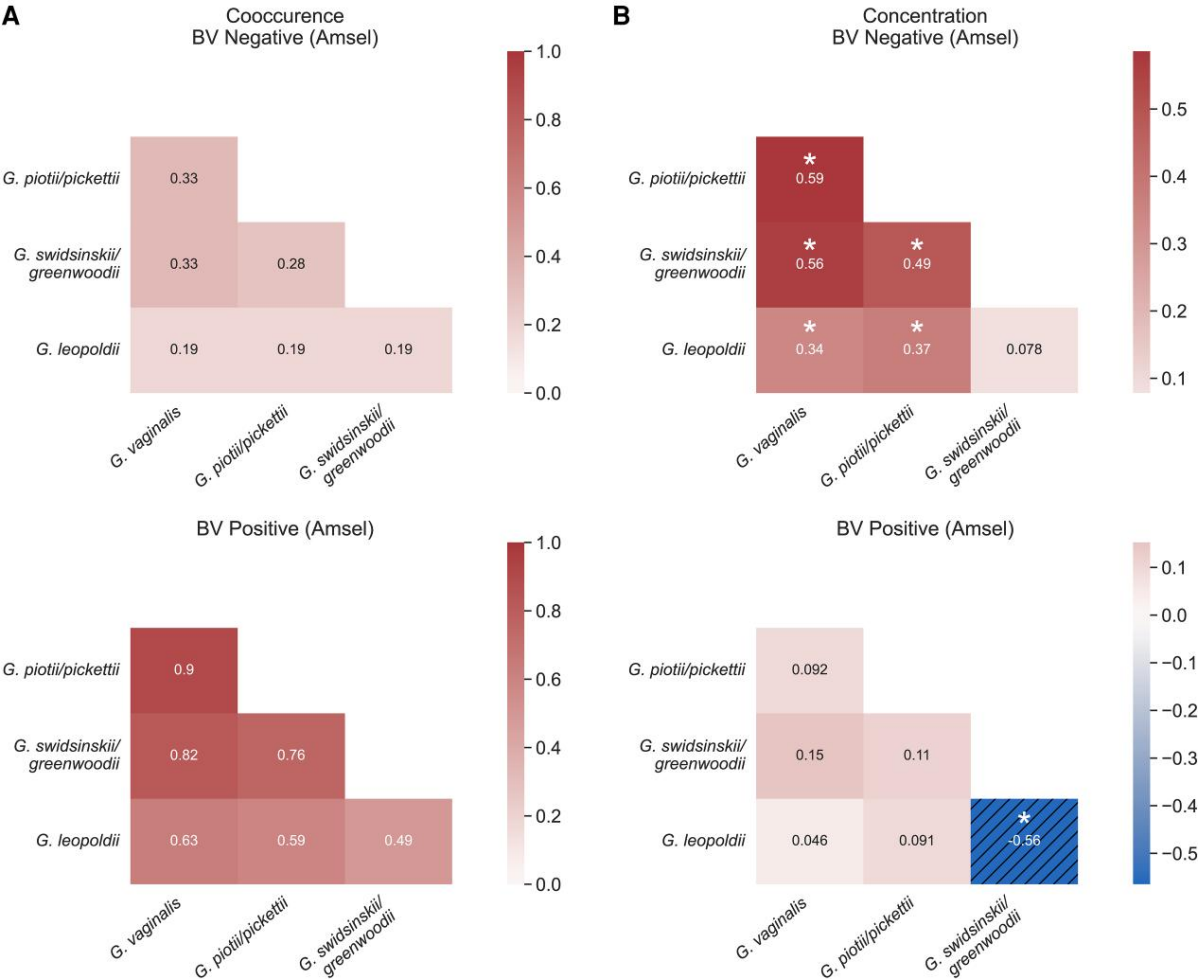
*Gardnerella* cooccurrence and correlation. *A*, Cooccurrence heatmaps of *Gardnerella* species groups in BV-negative (n = 150) and BV-positive (n = 101) participants by Amsel criteria. Percentage of BV-negative or BV-positive participants with cooccurrence of *Gardnerella* species group pairs by qPCR is shown with darker color indicating higher rate of cooccurrence. *B*, Spearman correlation values based on concentrations of each *Gardnerella* species group by qPCR in BV-negative (n = 150) and BV-positive (n = 101) participants by Amsel criteria. Correlation values are shown for each *Gardnerella* species group pair and colored according to correlation strength; solid color is positive correlation and hatch pattern is negative, with asterisks indicating statistical significance (*P* < .001). Abbreviations: BV, bacterial vaginosis; qPCR, quantitative polymerase chain reaction.

*Gardnerella* species groups were more frequently detected together in Amsel BV-positive participants than Amsel-negative participants (Figure 3*A*); 91.1% of participants with symptomatic BV had 3 or more *Gardnerella* species groups detected at baseline compared to 32.0% of BV-negative participants (*P* < .0001; Table 2). Most Amsel-positive participants had either all 4 *Gardnerella* species groups detected (46.5%) or *G. vaginalis*, *G. piotii/pickettii*, and *G. swidsinskii/greenwoodii* (29.7%). Of the 32.7% of Amsel-negative participants with 3 or more species groups detected, 45.8% had Nugent scores of 7–10 (BV) and 18.8% had Nugent scores of 4–6 (intermediate microbiota) (Supplementary Figure 2). Concentrations of *G. vaginalis*, *G. piotii/pickettii*, and G. *swidsinskii/greenwoodii* were all positively correlated with each other at baseline in BV-negative participants, but not in BV-positive participants (*P* < .0001). In those with BV, concentrations of *G. swidsinskii/greenwoodii* and *G. leopoldii* were negatively correlated (*P* < .001; Figure 3*B*). Study participants reporting a new sex partner within 60 days prior to baseline had significantly more species groups detected at baseline (2.6 vs 2.2, *P* = .03).

### *Gardnerella* Species and Subsequent Development of BV by Amsel Criteria

**Figure 4.**
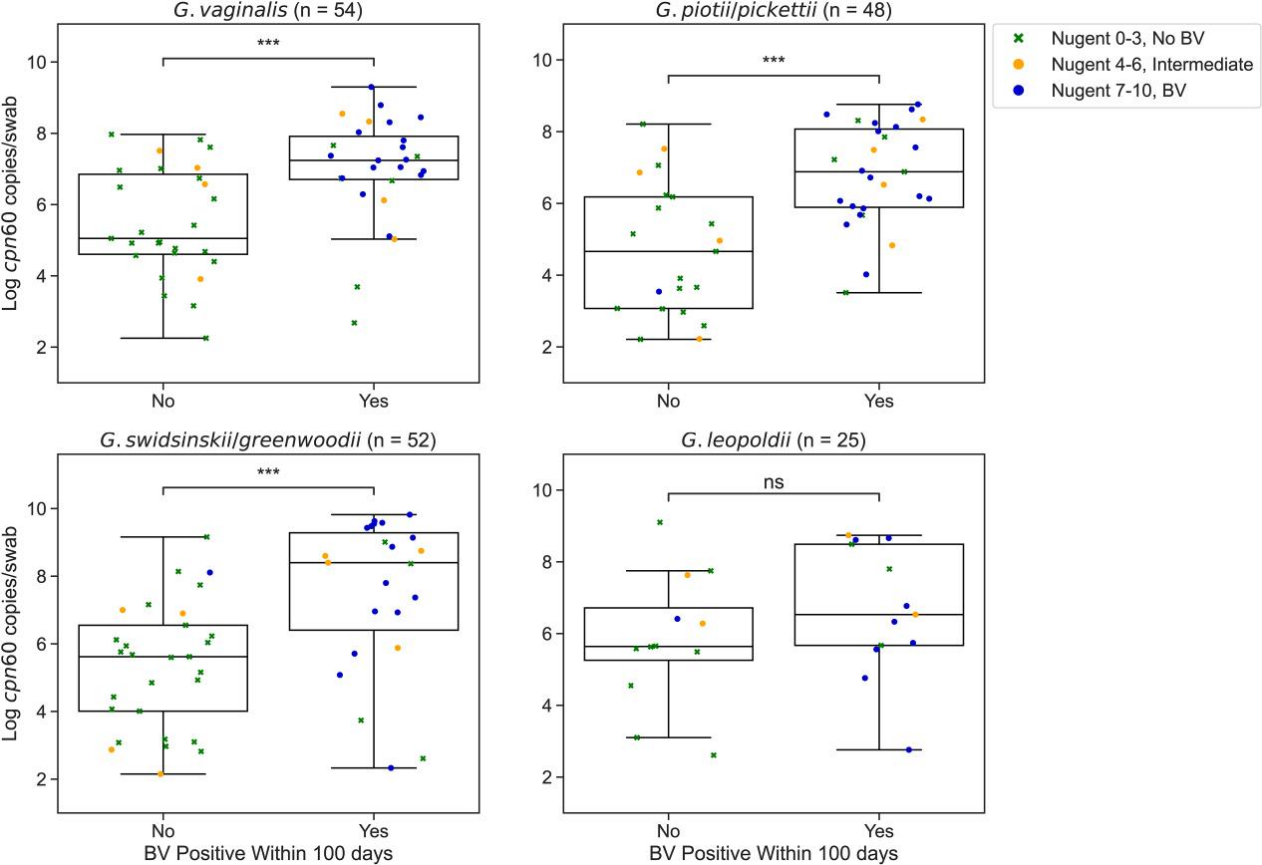
*Gardnerella cpn60* DNA concentrations in BV-negative participants at baseline and subsequent BV development. Log transformed baseline *Gardnerella cpn60* gene concentrations as measured by qPCR in BV-negative participants (Amsel criteria) with detection of each *Gardnerella* species group. Each point represents an individual participant and is colored and shaped based on Nugent score at baseline. Boxes show lower quartile, median, and upper quartile with whiskers extended to 1.5 times the interquartile range. Significance calculated using Mann-Whitney test. ***P < .001. Abbreviations: BV, bacterial vaginosis; ns, not significant; qPCR, quantitative polymerase chain reaction.

Only 3 of 81 (3.7%) Amsel-negative participants with 1 or more clinic follow-up visits and 2 or fewer *Gardnerella* species groups detected at baseline developed symptomatic BV at follow-up within 100 days (mean 55.3 [SD 30.9] days) compared to 26 of 43 (60.5%) Amsel-negative participants with 3 or more groups detected (53.7 [SD 25.1] days) (*P* < .0001). Participants with 3 or more *Gardnerella* species groups detected at baseline were also more likely to have reported a history of BV compared to participants with 2 or fewer groups detected (72.8% vs 48.6%). Median concentrations of *G. vaginalis* (7.24 vs 5.14 log_10_ copies/swab, *P* < .001), *G. piotii/pickettii* (6.88 vs 4.81 log_10_ copies/swab, *P* < .001), and *G. swidsinskii/greenwoodii* (8.40 vs 5.60 log_10_ copies/swab, *P* < .001) were significantly higher at baseline in Amsel-negative participants with follow-up who subsequently developed BV within 100 days compared to those who did not (Figure 4).

### *Gardnerella* Species Acquisition and Loss

In the 42 participants with weekly sampling data (16 Amsel positive, 26 Amsel negative), *G. vaginalis* and *G. swidsinskii/greenwoodii* were the most prevalent species groups at baseline (73.8% each), followed by *G. piotii/pickettii* (64.3%) and *G. leopoldii* (28.6%). *G. leopoldii* was the most frequently lost species with 75.0% of participants with detection at baseline having at least 1 weekly time point with concentrations below assay threshold, followed by *G. piotii/pickettii* (44.4%), *G. vaginalis* (35.5%), and *G. swidsinskii/greenwoodii* (29.0%). In participants without detection of a *Gardnerella* species group at baseline, *G. swidsinskii/greenwoodii* was the most acquired species (any weekly time point) (45.5%), followed by *G. leopoldii* (26.7%), *G. piotii/pickettii* (20.0%), and *G. vaginalis* (18.2%) (Supplementary Table 5). Instances of *G. swidsinskii/greenwoodii* acquisition or redetection were significantly higher in participants reporting vaginal intercourse on the same day or week prior (12 of 232 [5.2%] versus 4 of 256 [1.6%]; *P* = .04). We did not see statistically significant associations with acquisition or loss of other *Gardnerella* species groups and menses or sexual activity.

### Metronidazole Treatment Response

**Figure 5.**
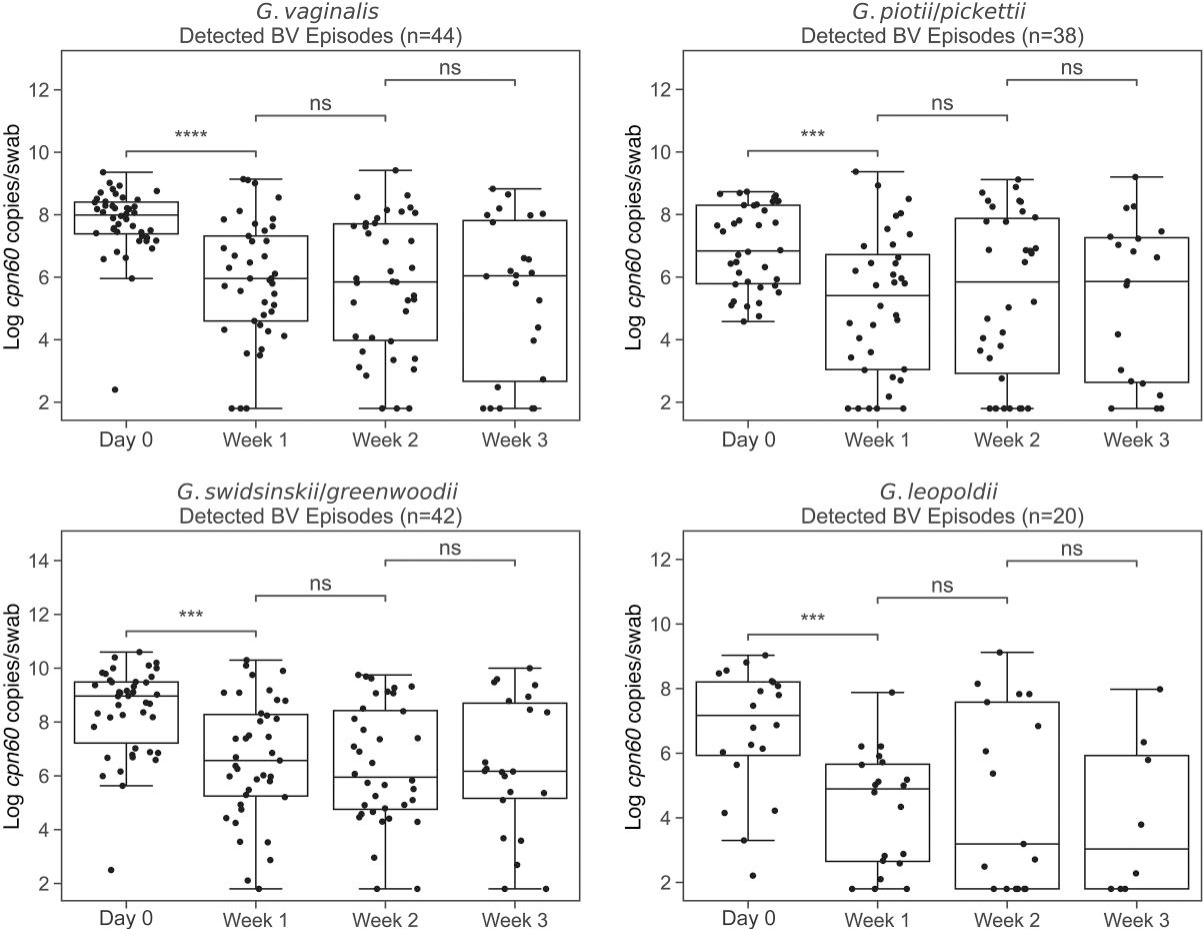
Changes in *Gardnerella* concentration in response to antibiotic treatment for symptomatic BV. Log transformed *Gardnerella* species group *cpn60* gene concentrations at antibiotic prescription (day 0), week 1 (days 1–7), week 2 (days 8–14), and week 3 (days 15–21). There were 44 episodes of BV and subsequent antibiotic prescription in 26 participants in the 12-week period examined. Only episodes with detection of each *Gardnerella* species group at prescription are shown in each plot. Boxes show lower quartile, median, and upper quartile with whiskers extended to 1.5 times the interquartile range. Mann-Whitney test was used to calculate significance. ***P < .001, ****P < .0001. Abbreviations: BV, bacterial vaginosis; ns, not significant.

There were 44 episodes of symptomatic BV with subsequent metronidazole treatment (24 episodes treated with metrogel once a day at bedtime for 5 days, 19 with metronidazole 500 mg orally twice a day for 7 days, and 1 with both) in 26 of 42 weekly longitudinal participants. Sixteen participants experienced additional or continuing episodes of symptomatic BV during the 12-week follow-up period and had more than 1 BV episode represented. *G. vaginalis*, *G. piotii/pickettii*, *G. swidsinskii/greenwoodii*, and *G. leopoldii* were detected in 44, 38, 42, and 20 BV episodes, respectively. Concentrations of each *Gardnerella* species group were significantly lower in the week after metronidazole treatment compared to prescription date (Figure 5). Of BV episodes, 8.9%, 23.7%, 4.8%, and 35.0% with *G. vaginalis*, *G. piotii/pickettii*, *G. swidsinskii/greenwoodii*, and G. *leopoldii* detection, respectively, resulted in no detection by week 2 postprescription, and redetection by week 3 occurred in 75.0%, 44.4%, 0.0%, and 28.6% of those episodes, respectively.

## DISCUSSION

The mechanisms underlying the onset of BV remain unclear and despite antibiotic treatment more than 50% of women may experience recurrent BV within a year of initial diagnosis [34]. *Gardnerella* is a prominent member of the vaginal microbiota and is almost always present in BV. BV pathogenesis models have been proposed that include *Gardnerell*a as a keystone species in BV development that may promote colonization by other BV-associated bacteria, and which may be sexually transmitted [8, 14]. However, the high prevalence of *Gardnerella* species in women without BV raises questions about whether *Gardnerella* is a true pathogen or an opportunist.

Currently, there are limited studies examining the roles of different *Gardnerella* species in the development of BV and most of these studies have utilized qPCR assays developed by Balashov et al targeting 4 previously defined *Gardnerella* clades, yielding varying results regarding which clades are associated with BV [10, 13, 21–24] (Supplementary Table 6). It has also been noted that these assays may not detect several more recently characterized clade 2 isolates [20]. Only 1 other published study to date has utilized species-specific *Gardnerella* qPCR assays targeting the *cpn60* gene [20]. The combination of varying study populations, use of Amsel criteria versus Nugent score for BV diagnosis, and potential underdetection of newly described *Gardnerella* species could all contribute to the heterogeneity of results from these studies.

In this study, we developed qPCR assays targeting the *cpn60* gene of 4 *Gardnerella* species groups and applied them to a cross-sectional set of vaginal swabs from study participants with and without BV, as well as a longitudinal set of weekly samples. We found increased prevalence and concentrations of all 4 *Gardnerella* species groups in participants with BV in addition to higher numbers of species groups detected, consistent with previous study results [10, 13, 21–24]. *G. vaginalis* was detected in 98.0% of participants with symptomatic BV but was also detected in 50.7% of Amsel-negative participants, although usually at lower concentrations. Despite being nearly always present in BV, *G. vaginalis* was always detected with at least 1 other *Gardnerella* species group at baseline in participants with symptomatic BV, suggesting that the presence of *G. vaginalis* alone may not be sufficient to facilitate BV. Most BV-positive participants had *G. vaginalis*, *G. piotii/pickettii*, and either 1 or both of *G. swidsinskii/greenwoodii* and *G. leopoldii* detected, although multiple *Gardnerella* species could also be detected for extended periods of time before symptomatic BV was diagnosed. Many participants who were Amsel negative with 3 or more *Gardnerella* species groups detected had Nugent scores indicative of BV or intermediate microbiota, and more than 60% of those participants with follow-up would develop symptomatic BV within 100 days compared to 3.7% of those with 2 or fewer *Gardnerella* species groups detected. These data suggest that vaginal colonization with multiple *Gardnerella* species is a risk factor for development of BV.

Interestingly, *G. swidsinskii/greenwoodii* and *G. leopoldii* were less frequently detected together than other *Gardnerella* species, consistent with findings from another study [19]. We also noted that concentrations of these 2 species groups were negatively correlated in BV-positive participants, and they were almost never exclusively detected together unless 1 or both of *G. vaginalis* or *G. piotii/pickettii* was also detected. Past studies utilizing qPCR to quantify *Gardnerella* clades or species have not differentiated between *G. swidsinskii* and *G. leopoldii* [10, 13, 20–24]. These 2 species were previously classified within the same clade, but matrix-assisted laser desorption ionization time-of-flight (MALDI-TOF) and whole-genome sequencing have determined these to be separate species [17]. A previous study comparing these 2 species noted the appearance of a polysaccharide-like capsule in *G. swidsinskii* that was absent in *G. leopoldii* as well as differences in cytotoxicity and epithelial cell adhesion in vitro [35]. *G. leopoldii* was the least prevalent *Gardnerella* species group detected in our study population and was frequently lost in our longitudinal cohort, suggesting it may be a more transient member of the vaginal microbiota. Interestingly, *G. swidsinskii/greenwoodii* was the most frequently acquired species group, and acquisition events were significantly higher in participants reporting vaginal intercourse in the prior week, although this was driven by low sample numbers. More research is needed to explore how *G. leopoldii* and *G. swidsinskii* may be interacting in the vagina (such as with competitive exclusion) and whether *G. swidsinskii* is more likely to be sexually transmitted.

Overall, our results suggest that BV is associated with increased *Gardnerella* diversity and concentration, and do not suggest that these 4 *Gardnerella* species groups are specific for BV, although the existence of other, more virulent *Gardnerella* strains is possible as others have suggested [14, 15, 36]. The presence of multiple *Gardnerella* species was necessary for symptomatic BV to manifest in our study population. This could explain high rates of *Gardnerella* detection in healthy women and complicates the notion that *Gardnerella* is a keystone species in BV. *Gardnerella* has long been associated with biofilm formation, which may facilitate growth of BV-associated anaerobes such as *Fannyhessea vaginae* and *Prevotella bivia* [12, 37–39]. Biofilm formation by *Gardnerella* has been shown to increase in the higher pH range typically found in BV and may be suppressed by *Lactobacillus* species found in an optimal, non-BV state [40]. Variation in biofilm forming potential has been observed between *Gardnerella* isolates in vitro [36] and *Gardnerella* isolates are known to possess different virulence factors, such as sialidase and vaginolysin, which may uniquely contribute to BV development [41–43]. Acquisition of new *Gardnerella* species through sexual activity or other means could promote biofilm formation and growth of other BV-associated bacteria, leading to development of BV. However, a previous study did not see increased biofilm formation in cocultures of multiple *Gardnerella* clades in vitro compared to *Gardnerella* monocultures [44], although these cultures did not include any additional BV-associated bacteria such as *F. vaginae* that may contribute to biofilm formation. There have been no published studies to date exploring virulence potential in cocultures of multiple *Gardnerella* species.

In the 44 symptomatic BV episodes from our longitudinal cohort, *Gardnerella* concentrations of all 4 species groups were significantly lower in the week after metronidazole prescription for BV. However, detection below threshold of any *Gardnerella* species group by week 2 was uncommon, and redetection could occur within a week. Previous studies have shown that some *Gardnerella* strains are resistant to metronidazole [45, 46], and biofilm formation has also been shown to increase metronidazole and clindamycin resistance of *Gardnerella* [47, 48]. This limited activity of metronidazole against *Gardnerella* may contribute to BV recurrence, and more effective treatments that specifically target *Gardnerella* may be needed to improve treatment outcomes.

There are several limitations to this study including our ability to detect only 4 species groups within the *Gardnerella* genus via qPCR. We were unable to differentiate between *G. vaginalis* and genomospecies 2 nor between *G. piotii* and *G. pickettii* via *cpn60* qPCR, although previous studies have placed these species in the same clade and subgroup [49, 50]. Likewise, we were unable to completely differentiate between *G. swidsinskii, G. greenwoodii,* and genomospecies 9 and 10, which were nevertheless detected with reduced sensitivity. Previous studies have placed *G. greenwoodii* and genomospecies 9 and 10 in subgroup D or clade 3 [49, 50], and it has been observed that this clade is not as prevalent nor abundant as the other 3 clades [13, 19, 22], although at least 1 study observed increased growth of subgroup D in the presence of other subgroups in vitro [44]. Additionally, our assays did not detect *Gardnerella* genomospecies 7, which has been associated with refractory BV following oral metronidazole treatment and may play a role in BV development in some women [20]. In the longitudinal cohort, weekly sampling and incomplete or minimal behavioral data, including antibiotic use other than metronidazole, limited our ability to associate acquisition and loss of *Gardnerella* species groups with specific behaviors. Lastly, participants enrolled in this study were more likely to have had a history of BV and this may have influenced our results through higher rates of *Gardnerella* detection.

Many questions remain regarding diversity within the *Gardnerella* genus and its contributions to vaginal health. Additional studies exploring the *Gardnerella* pangenome as well as in vitro characterization of different *Gardnerella* species and isolates will be necessary to determine the genotypic and phenotypic variation within the genus. Given the increased *Gardnerella* diversity seen in BV, development and utilization of molecular techniques to differentiate *Gardnerella* species will be vital in understanding the roles these species play in BV etiology. Unraveling the dynamics of competition and cooperation between different *Gardnerella* species, BV-associated anaerobes, and lactobacilli may lead to interventions that promote vaginal health.

## Supplementary Data

Supplementary materials are available at *The Journal of Infectious Diseases* online (http://jid.oxfordjournals.org/). Supplementary materials consist of data provided by the author that are published to benefit the reader. The posted materials are not copyedited. The contents of all supplementary data are the sole responsibility of the authors. Questions or messages regarding errors should be addressed to the author.

## Supplementary Material

jiae026_Supplementary_Data

## Notes

***Author contributions*.** D. N. F. and S. S. designed the study. M. M., S. M. S., and T. F. performed laboratory work. M. M., S. P., S. M. S., and T. F. assisted in data curation and analysis. M. M. and D. N. F. prepared the manuscript. M. M. and S. P. created graphical figures. All authors contributed to manuscript review and editing.

***Acknowledgments.*** We thank the clinicians and study coordinators who assisted with study enrollment and sample collection, as well as all study participants who contributed to the study. We also thank D. J. Valint and Congzhou Liu for their assistance with DNA extraction of samples.

***Data availability.*** Data available upon request.

***Financial support*.** This work was supported by the National Institutes of Health (grant number R01 AI061628 to D. N. F.). Funding to pay the Open Access publication charges for this article was provided by the Fred Hutchinson Cancer Center.

## References

[1] Peebles K , Velloza J, Balkus JE, McClelland RS, Barnabas RV. High global burden and costs of bacterial vaginosis: a systematic review and meta-analysis. Sex Transm Dis 2019; 46:304–11. 30624309 10.1097/OLQ.0000000000000972

[2] Fredricks DN , Fiedler TL, Marrazzo JM. Molecular identification of bacteria associated with bacterial vaginosis. N Engl J Med 2005; 353:1899–911. 16267321 10.1056/NEJMoa043802

[3] Srinivasan S , Hoffman NG, Morgan MT, et al Bacterial communities in women with bacterial vaginosis: high resolution phylogenetic analyses reveal relationships of microbiota to clinical criteria. PLoS One 2012; 7:e37818. 22719852 10.1371/journal.pone.0037818 PMC3377712

[4] Hillier SL , Nugent RP, Eschenbach DA, et al Association between bacterial vaginosis and preterm delivery of a low-birth-weight infant. The vaginal infections and prematurity study Group. N Engl J Med 1995; 333:1737–42. 7491137 10.1056/NEJM199512283332604

[5] Atashili J , Poole C, Ndumbe PM, Adimora AA, Smith JS. Bacterial vaginosis and HIV acquisition: a meta-analysis of published studies. AIDS 2008; 22:1493–501. 18614873 10.1097/QAD.0b013e3283021a37 PMC2788489

[6] Cherpes TL , Meyn LA, Krohn MA, Lurie JG, Hillier SL. Association between acquisition of herpes simplex virus type 2 in women and bacterial vaginosis. Clin Infect Dis 2003; 37:319–25. 12884154 10.1086/375819

[7] Haggerty CL , Hillier SL, Bass DC, Ness RB; PID Evaluation and Clinical Health (PEACH) Study Investigators. Bacterial vaginosis and anaerobic bacteria are associated with endometritis. Clin Infect Dis 2004; 39:990–5. 15472851 10.1086/423963

[8] Muzny CA , Schwebke JR. Pathogenesis of bacterial vaginosis: discussion of current hypotheses. J Infect Dis 2016; 214(Suppl 1):S1–5. 27449868 10.1093/infdis/jiw121 PMC4957507

[9] Gardner HL , Dukes CD. *Haemophilus vaginalis* vaginitis: a newly defined specific infection previously classified non-specific vaginitis. Am J Obstet Gynecol 1955; 69:962–76. 14361525

[10] Balashov SV , Mordechai E, Adelson ME, Gygax SE. Identification, quantification and subtyping of *Gardnerella vaginalis* in noncultured clinical vaginal samples by quantitative PCR. J Med Microbiol 2014; 63:162–75. 24200640 10.1099/jmm.0.066407-0

[11] Cox C , McKenna JP, Watt AP, Coyle PV. New assay for *Gardnerella vaginalis* loads correlates with Nugent scores and has potential in the diagnosis of bacterial vaginosis. J Med Microbiol 2015; 64:978–84. 26296660 10.1099/jmm.0.000118

[12] Swidsinski A , Mendling W, Loening-Baucke V, et al Adherent biofilms in bacterial vaginosis. Obstet Gynecol 2005; 106:1013–23. 16260520 10.1097/01.AOG.0000183594.45524.d2

[13] Plummer EL , Vodstrcil LA, Murray GL, et al *Gardnerella vaginalis* clade distribution is associated with behavioral practices and Nugent score in women who have sex with women. J Infect Dis 2020; 221:454–63. 31544206 10.1093/infdis/jiz474

[14] Muzny CA , Taylor CM, Swords WE, et al An updated conceptual model on the pathogenesis of bacterial vaginosis. J Infect Dis 2019; 220:1399–405. 31369673 10.1093/infdis/jiz342 PMC6761952

[15] Morrill S , Gilbert NM, Lewis AL. *Gardnerella vaginalis* as a cause of bacterial vaginosis: appraisal of the evidence from in vivo models. Front Cell Infect Microbiol 2020; 10:168. 32391287 10.3389/fcimb.2020.00168 PMC7193744

[16] Hickey RJ , Forney LJ. *Gardnerella vaginalis* does not always cause bacterial vaginosis. J. Infect. Dis 2014; 210:1682–3. 24855684 10.1093/infdis/jiu303 PMC4334793

[17] Vaneechoutte M , Guschin A, Van Simaey L, Gansemans Y, Van Nieuwerburgh F, Cools P. Emended description of *Gardnerella vaginalis* and description of *Gardnerella leopoldii* sp. Nov., *Gardnerella piotii* sp. Nov. and *Gardnerella swidsinskii* sp. Nov., with delineation of 13 genomic species within the genus *Gardnerella*. Int J Syst Evol Microbiol 2019; 69:679–87. 30648938 10.1099/ijsem.0.003200

[18] Sousa M , Ksiezarek M, Perovic SU, et al *Gardnerella pickettii* sp. Nov. (formerly *Gardnerella* genomic species 3) and *Gardnerella greenwoodii* sp. Nov. (formerly *Gardnerella* genomic species 8) isolated from female urinary microbiome. Int J Syst Evol Microbiol 2023; 73:006140. 10.1099/ijsem.0.006140 37921436

[19] Hill JE , Albert AYK. Resolution and cooccurrence patterns of *Gardnerella leopoldii*, *G. swidsinskii*, *G. piotii*, and *G. vaginalis* within the vaginal microbiome. Infect Immun 2019; 87:e00532-19. 31527125 10.1128/IAI.00532-19 PMC6867840

[20] Turner E , Sobel JD, Akins RA. Prognosis of recurrent bacterial vaginosis based on longitudinal changes in abundance of *Lactobacillus* and specific species of *Gardnerella*. PLoS One 2021; 16:e0256445. 34424942 10.1371/journal.pone.0256445 PMC8382169

[21] Janulaitiene M , Paliulyte V, Grinceviciene S, et al Prevalence and distribution of *Gardnerella vaginalis* subgroups in women with and without bacterial vaginosis. BMC Infect Dis 2017; 17:394. 28583109 10.1186/s12879-017-2501-y PMC5460423

[22] Shipitsyna E , Krysanova A, Khayrullina G, et al Quantitation of all four *Gardnerella vaginalis* clades detects abnormal vaginal microbiota characteristic of bacterial vaginosis more accurately than putative *G. vaginalis* sialidase A gene count. Mol Diagn Ther 2019; 23:139–47. 30721449 10.1007/s40291-019-00382-5 PMC6394432

[23] Hilbert DW , Schuyler JA, Adelson ME, Mordechai E, Sobel JD, Gygax SE. *Gardnerella vaginalis* population dynamics in bacterial vaginosis. Eur J Clin Microbiol Infect Dis 2017; 36:1269–78. 28197729 10.1007/s10096-017-2933-8

[24] Vodstrcil LA , Twin J, Garland SM, et al The influence of sexual activity on the vaginal microbiota and *Gardnerella vaginalis* clade diversity in young women. PLoS One 2017; 12:e0171856. 28234976 10.1371/journal.pone.0171856 PMC5325229

[25] Fredricks DN , Plantinga A, Srinivasan S, et al Vaginal and extra-vaginal bacterial colonization and risk for incident bacterial vaginosis in a population of women who have sex with men. J Infect Dis 2022; 225:1261–5. 32379324 10.1093/infdis/jiaa233 PMC8974833

[26] Amsel R , Totten PA, Spiegel CA, Chen KCS, Eschenbach D, Holmes KK. Nonspecific vaginitis: diagnostic criteria and microbial and epidemiologic associations. Am J Med 1983; 74:14–22. 6600371 10.1016/0002-9343(83)91112-9

[27] Nugent RP , Krohn MA, Hillier SL. Reliability of diagnosing bacterial vaginosis is improved by a standardized method of gram stain interpretation. J Clin Microbiol 1991; 29:297–301. 1706728 10.1128/jcm.29.2.297-301.1991 PMC269757

[28] Fredricks DN , Fiedler TL, Thomas KK, Mitchell CM, Marrazzo JM. Changes in vaginal bacterial concentrations with intravaginal metronidazole therapy for bacterial vaginosis as assessed by quantitative PCR. J Clin Microbiol 2009; 47:721–6. 19144794 10.1128/JCM.01384-08 PMC2650913

[29] Hunter JD . Matplotlib: a 2D graphics environment. Comput Sci Eng 2007; 9:90–5.

[30] Waskom ML . Seaborn: statistical data visualization. J Open Source Softw 2021; 6:3021.

[31] Charlier F , Weber M, Izak D, et al trevismd/statannotations: v0.5. Zenodo. 2022. https://zenodo.org/record/7213391. Accessed 31 July 2023.

[32] R Core Team . R: A language and environment for statistical computing. R Foundation for Statistical Computing, Vienna, Austria, 2023. https://www.R-project.org/. Accessed 31 July 2023.

[33] Revelle W . psych: procedures for psychological, psychometric, and personality research. R package version 2.3.3, 2023. https://CRAN.R-project.org/package=psych. Accessed 31 July 2023.

[34] Bradshaw CS , Morton AN, Hocking J, et al High recurrence rates of bacterial vaginosis over the course of 12 months after oral metronidazole therapy and factors associated with recurrence. J Infect Dis 2006; 193:1478–86. 16652274 10.1086/503780

[35] Harwich MD , Alves JM, Buck GA, et al Drawing the line between commensal and pathogenic *Gardnerella vaginalis* through genome analysis and virulence studies. BMC Genomics 2010; 11:375. 20540756 10.1186/1471-2164-11-375 PMC2890570

[36] Janulaitiene M , Gegzna V, Baranauskiene L, Bulavaitė A, Simanavicius M, Pleckaityte M. Phenotypic characterization of *Gardnerella vaginalis* subgroups suggests differences in their virulence potential. PLoS One 2018; 13:e0200625. 30001418 10.1371/journal.pone.0200625 PMC6042761

[37] Patterson JL , Stull-Lane A, Girerd PH, Jefferson KK. Analysis of adherence, biofilm formation and cytotoxicity suggests a greater virulence potential of *Gardnerella vaginalis* relative to other bacterial-vaginosis-associated anaerobes. Microbiology 2010; 156:392–9. 19910411 10.1099/mic.0.034280-0 PMC2890091

[38] Castro J , Alves P, Sousa C, et al Using an in-vitro biofilm model to assess the virulence potential of bacterial vaginosis or non-bacterial vaginosis *Gardnerella vaginalis* isolates. Sci Rep 2015; 5:11640. 26113465 10.1038/srep11640 PMC4481526

[39] Rosca AS , Castro J, França Â, Vaneechoutte M, Cerca N. *Gardnerella vaginalis* dominates multi-species biofilms in both pre-conditioned and competitive in vitro biofilm formation models. Microb Ecol 2022; 84:1278–87. 34741647 10.1007/s00248-021-01917-2

[40] He Y , Na R, Niu X, Xiao B, Yang H. *Lactobacillus rhamnosus* and *Lactobacillus casei* affect various stages of *Gardnerella* species biofilm formation. Front Cell Infect Microbiol 2021; 11:568178. 33680986 10.3389/fcimb.2021.568178 PMC7933028

[41] Robinson LS , Schwebke J, Lewis WG, Lewis AL. Identification and characterization of NanH2 and NanH3, enzymes responsible for sialidase activity in the vaginal bacterium *Gardnerella vaginalis*. J Biol Chem 2019; 294:5230–45. 30723162 10.1074/jbc.RA118.006221 PMC6462536

[42] Kurukulasuriya SP , Patterson MH, Hill JE. Slipped-strand mispairing in the gene encoding sialidase NanH3 in *Gardnerella* spp. Infect Immun 2021; 89:e00583-20. 33361200 10.1128/IAI.00583-20 PMC8097274

[43] Garcia EM , Serrano MG, Edupuganti L, Edwards DJ, Buck GA, Jefferson KK. Sequence comparison of vaginolysin from different *Gardnerella* species. Pathogens 2021; 10:86. 33498226 10.3390/pathogens10020086 PMC7909246

[44] Khan S , Voordouw MJ, Hill JE. Competition among *Gardnerella* subgroups from the human vaginal microbiome. Front Cell Infect Microbiol 2019; 9:374. 31737577 10.3389/fcimb.2019.00374 PMC6834547

[45] Schuyler JA , Mordechai E, Adelson ME, Sobel JD, Gygax SE, Hilbert DW. Identification of intrinsically metronidazole-resistant clades of *Gardnerella vaginalis*. Diagn Microbiol Infect Dis 2016; 84:1–3. 26514076 10.1016/j.diagmicrobio.2015.10.006

[46] Landlinger C , Oberbauer V, Tisakova LP, et al Preclinical data on the *Gardnerella*-specific endolysin PM-477 indicate its potential to improve the treatment of bacterial vaginosis through enhanced biofilm removal and avoidance of resistance. Antimicrob Agents Chemother 2022; 66:e0231921. 35416708 10.1128/aac.02319-21 PMC9112913

[47] Gottschick C , Szafranski SP, Kunze B, et al Screening of compounds against *Gardnerella vaginalis* biofilms. PLoS One 2016; 11:e0154086. 27111438 10.1371/journal.pone.0154086 PMC4844189

[48] Li T , Zhang Z, Wang F, et al Antimicrobial susceptibility testing of metronidazole and clindamycin against *Gardnerella vaginalis* in planktonic and biofilm formation. Can J Infect Dis Med Microbiol 2020; 2020:1361825. 32612729 10.1155/2020/1361825 PMC7315270

[49] Ahmed A , Earl J, Retchless A, et al Comparative genomic analyses of 17 clinical isolates of *Gardnerella vaginalis* provide evidence of multiple genetically isolated clades consistent with subspeciation into genovars. J Bacteriol 2012; 194:3922–37. 22609915 10.1128/JB.00056-12 PMC3416530

[50] Paramel Jayaprakash T , Schellenberg JJ, Hill JE. Resolution and characterization of distinct *cpn60*-based subgroups of *Gardnerella vaginalis* in the vaginal microbiota. PLoS One 2012; 7:e43009. 22900080 10.1371/journal.pone.0043009 PMC3416817

