# Supplementary material for: Gardnerella Species and Their Association With Bacterial Vaginosis
Source: J Infect Dis. 2024 Jan 24;230(1):e171–81. doi: 10.1093/infdis/jiae026 (PMC11272073; doi:10.1093/infdis/jiae026)
Supplement: jiae026_Supplementary_Data [file jiae026_supplementary_data.zip › supp_methods.docx]

**Supplementary Methods**

**Study Population and Sample Collection**

From October 2012 to August 2015, vaginal swabs were collected from 251 study participants with (n=101) and without (n=150) BV attending the Public Health–Seattle & King County Sexual Health Clinic. The overall study population included individuals assigned female sex at birth in Seattle, Washington with and without a history of BV. Study participants in a risk group for BV based on sexual behavior (increased number of sex partners, male or female), history of BV, or race/ethnicity (identifying as Black or Hispanic) were also included. No study participants were pregnant at enrollment and NAATs were performed to screen for *Chlamydia trachomatis*, *Neisseria gonorrhoeae*, and *Trichomonas vaginalis*. HIV testing was not performed. The study was approved by the Fred Hutchinson Cancer Center IRB (IRB Protocol #7683) and all study participants provided written, informed consent.

Vaginal swabs were collected by clinicians at baseline and any subsequent follow up clinic visits and stored at -80°C. Study participants had regularly scheduled follow up visits approximately every 30 days, when experiencing BV symptoms, or 7-14 days from a BV positive visit for follow up. Study participants were assessed for BV at each clinic visit by Amsel criteria, and vaginal smears of all clinic samples were prepared on glass slides for Gram stain Nugent scoring [1]. Most study participants did not remain enrolled for more than 100 days, and this was used as a cutoff for BV development analysis in this study. Participants were considered BV positive when at least 3 of 4 Amsel clinical criteria were met (symptomatic BV) [2]. Participants with symptomatic BV were treated with metronidazole at all clinic visits according to CDC guidelines with either metrogel qHS for 5 days and/or 500 mg metronidazole po bid for 7 days.

A subset of 42 participants provided at least 10 weekly self-collected vaginal swabs for up to 12 weeks from baseline, and these samples were used for longitudinal analysis. These participants were selected for longitudinal analysis as they previously had swabs extracted for DNA as part of prior analyses and had at least 10 weekly swabs available in the 12 weeks after baseline enrollment. Self-collected swabs were stored up to 30 days by study participants in home freezers before being mailed or brought into clinic, either frozen or at room temperature. Swabs were stored at -80°C upon receipt. We targeted swabs collected every 7 days after baseline, but swabs collected up to 3 days before or after each weekly target date were used when weekly target date swabs were unavailable (i.e., if a day 7 swab was unavailable for week 1, swabs from days 4-10 could be utilized, prioritizing swabs closest to the target date). Clinic swabs collected at follow up visits within the 12-week period were also used when available. Data on participant sexual behavior, vaginal product use, antibiotic use, and menses were collected via questionnaires at clinic visits and via self-reported daily diaries.

**DNA Extraction**

Swabs were thawed on ice and mixed with 500 µL filtered 0.9% saline before pelleting for DNA extraction using the Qiagen QIAamp BiOstic Bacteremia DNA Kit (Hilden, Germany; formerly from Mo Bio Laboratories). DNA was eluted in 75 µL of EB buffer and 75 µL of filtered 0.2X Tris-EDTA. To increase DNA extraction throughput, a subset of 35 baseline samples (33 Amsel positive, 2 Amsel negative) and 48 longitudinal samples from 4 Amsel negative participants were extracted using the Qiagen PowerFecal DNA Kit on an automated Qiagen QIAcube and eluted in 150-200 µL buffer. We did not see a significant difference in *Gardnerella* concentrations from samples extracted via the PowerFecal DNA Kit compared to the QIAamp BiOstic Bacteremia DNA kit when adjusted for BV status. Sham extraction control swabs were extracted in the same way as study swabs to monitor for contamination during DNA extraction and qPCR.

**qPCR**

PCR inhibition was measured using an internal amplification control qPCR assay [3] and total bacterial DNA was estimated using a broad-range qPCR assay targeting the 16S rRNA gene as previously described [4]. For *Gardnerella* cpn60 qPCR assays, 15 µL reactions were run on a QuantStudio™ 6 Flex Real-Time PCR System for 45 cycles using 1X TaqMan™ Fast Advanced Master Mix, 0.8 or 1.2 µM primer, and 150 nM probe (see Supplementary Table 1 for oligonucleotide sequences and assay conditions). Specificity testing for the developed assays was performed using genomic DNA from 56 *Gardnerella* isolates (including representatives of each of the 13 *Gardnerella* genomospecies and type strains ATCC 14018 (*G. vaginalis*), CCUG 72427 (*G. piotii*), CCUG 72429 (*G. swidsinskii*), and CCUG 72425 (*G. leopoldii*)), 51 non-*Gardnerella* isolates representative of the vaginal microbiota, and Promega human genomic DNA (Madison, WI, USA). The limit of detection for each assay was 62.5 gene copies per swab.

**References**

1. Nugent RP, Krohn MA, Hillier SL. Reliability of diagnosing bacterial vaginosis is improved by a standardized method of gram stain interpretation. J Clin Microbiol. **1991**; 29(2):297–301.
2. Amsel R, Totten PA, Spiegel CA, Chen KCS, Eschenbach D, Holmes KK. Nonspecific vaginitis: Diagnostic criteria and microbial and epidemiologic associations. Am J Med. 1983; 74(1):14–22.
3. Khot PD, Ko DL, Hackman RC, Fredricks DN. Development and optimization of quantitative PCR for the diagnosis of invasive aspergillosis with bronchoalveolar lavage fluid. BMC Infect Dis. **2008**; 8(1):1–13.
4. Srinivasan S, Hoffman NG, Morgan MT, et al. Bacterial Communities in Women with Bacterial Vaginosis: High Resolution Phylogenetic Analyses Reveal Relationships of Microbiota to Clinical Criteria. PLoS One. **2012**; 7(6):1–15.
