# Supplementary material for: Gardnerella Species and Their Association With Bacterial Vaginosis
Source: J Infect Dis. 2024 Jan 24;230(1):e171–81. doi: 10.1093/infdis/jiae026 (PMC11272073; doi:10.1093/infdis/jiae026)
Supplement: jiae026_Supplementary_Data [file jiae026_supplementary_data.zip › supp_table1.docx]

**Supplementary Table 1|** *Gardnerella* cpn60 qPCR Assay Primers, Probes, and Conditions

| **Assay** | **Primers** | **Probe** | **PCR Conditions** |
| --- | --- | --- | --- |
| *Gardnerella vaginalis* and  *Gardnerella* genomospecies 2 ^a^ | F 5’-CTAAGGTTGAGAAGTCTGCT-3’  R 5’-CTTCTACAGCACGGAACACA-3’  0.8 µM | 5’-FAM-TGTTGCTCTTAGTGGAGAAGAAGCC  -TAM-3’  150 nM | 55°C Anneal, 20 sec  72°C Extend, 20 sec |
| *Gardnerella piotii* and *Gardnerella*  pickettii ^b^ | F1 5’-CAAAGGTTGAGAAGTCTCAA-3’  F2 5’-AAAGGTTGAGAAGTCTGCC-3’  R 5’-TCTCCAGAAACACCGCTA-3’  1.2 µM | 5’-FAM-  CGCAAATCTTAAGGGCGAAGAAGCT  -TAM-3’  150 nM | 57°C Anneal, 20 sec  72°C Extend, 20 sec |
| *Gardnerella swidsinskii* and *Gardnerella greenwoodii ^c^*  *Gardnerella* genomospecies 9 and 10 detected with reduced sensitivity ^d^ | F 5’-CTGCAAAGATTGAAAAGTCTCA-3’  R 5’-ATCTCCAGAAACGCCAGAG-3’  0.8 µM | 5’-FAM-  AATCACAGAGCTTAAGGGYGAA  -MGB-NFQ-3’  150 nM | 57°C Anneal, 20 sec  72°C Extend, 20 sec |
| *Gardnerella leopoldii* | F 5’-TGCGAAGATTGAAAAGACACC-3’  R 5’-GTCTCCAGAAACACCAGAG-3’  0.8 µM | 5’-FAM-  TATCACAGAGCTTAAGGGTGAA  -MGB-NFQ-3’  150 nM | 57°C Anneal, 20 sec  72°C Extend, 20 sec |

^a^*Gardnerella* genomospecies 2 detected with 80.5% efficiency compared to *G. vaginalis.*

^b^*G. pickettii* detected with 41.7% efficiency compared to *G. piotii.*

^c^*G. greenwoodii* detected with 53.7% efficiency compared to *G. swidsinskii* (differential amplification possible depending on *G. greenwoodii* strain type used).

*^d^Gardnerella* genomospecies 9 and 10 detected with 3.95e-04%, and 4.43e-02% efficiency respectively compared to *G. swidsinskii*.
