# Supplementary material for: Gardnerella Species and Their Association With Bacterial Vaginosis
Source: J Infect Dis. 2024 Jan 24;230(1):e171–81. doi: 10.1093/infdis/jiae026 (PMC11272073; doi:10.1093/infdis/jiae026)
Supplement: jiae026_Supplementary_Data [file jiae026_supplementary_data.zip › supp_table2.docx]

**Supplementary Table 2|** Additional participant characteristics at baseline

|  | **All Participants** | **BV Negative (Amsel)** | **BV Positive (Amsel)** | **Longitudinal Participants** | **Longitudinal BV Negative (Amsel)** | **Longitudinal**  **BV Positive (Amsel)** |
| --- | --- | --- | --- | --- | --- | --- |
| **N** | 251 | 150 | 101 | 42 | 26 | 16 |
|  |  |  |  |  |  |  |
| **Amsel Criteria** |  |  |  |  |  |  |
| pH (Median ± SD, Range) | 4.9 ± 0.51, 4.0-6.5 | 4.4 ± 0.51, 4.0-6.5 | 5.0 ± 0.31, 4.7-6.5 | 5.0 ± 0.49,  4.0-6.5 | 4.7 ± 0.55,  4.0-6.5 | 5.1 ± 0.23,  4.7-5.5 |
| Whiff Test Positive | 88 (35.1%) | 2 (1.3%) | 86 (85.1%) | 14 (33.3%) | 0 (0%) | 14 (87.5%) |
| Thin, homogenous discharge | 76 (30.3%) | 4 (2.7%) | 72 (71.3%) | 11 (26.2%) | 1 (3.8%) | 10 (62.5%) |
| Clue Cells | 129 (51.4%) | 29 (19.3%) | 100 (99.0%) | 24 (57.1%) | 8 (30.8%) | 16 (100%) |
|  |  |  |  |  |  |  |
| **Yeast/STIs** |  |  |  |  |  |  |
| Yeast | 9 (3.5%) | 6 (4.0%) | 3 (3.0%) | 1 (2.4%) | 0 (0%) | 1 (6.3%) |
| GC | 1 (0.4%) | 1 (0.7%) | 0 (0%) | 0 (0%) | 0 (0%) | 0 (0%) |
| NC | 2 (0.8%) | 2 (1.3%) | 0 (0%) | 1 (2.4%) | 1 (3.8%) | 0 (0%) |
| Trichomonas | 12 (4.8%) | 6 (4.0%) | 6 (5.9%) | 1 (2.4%) | 1 (3.8%) | 0 (0%) |
|  |  |  |  |  |  |  |
| **Oral Antibiotics**  **Last 30 Days** |  |  |  |  |  |  |
| Yes | 27 (10.8%) | 18 (12.0%) | 9 (8.9%) | 4 (9.5%) | 4 (15.4%) | 0 (0%) |
| No | 220 (87.6%) | 129 (86.0%) | 91 (90.1%) | 36 (85.7%) | 21 (80.8%) | 15 (93.8%) |
| Don't Know or No Response | 4 (1.6%) | 3 (2.0%) | 1 (1.0%) | 2 (4.8%) | 1 (3.8%) | 1 (6.3%) |
|  |  |  |  |  |  |  |
| **Vaginal Antibiotics**  **Last 30 Days** |  |  |  |  |  |  |
| Yes | 15 (6.0%) | 9 (6.0%) | 6 (5.9%) | 3 (7.1%) | 2 (7.7%) | 1 (6.3%) |
| No | 233 (92.8%) | 140 (93.3%) | 93 (92.1%) | 38 (90.5%) | 24 (92.3%) | 14 (87.5%) |
| Don't Know or No Response | 3 (1.2%) | 1 (0.7%) | 2 (2.0%) | 1 (2.4%) | 0 (0%) | 1 (6.3%) |
|  |  |  |  |  |  |  |
| **Vaginal Nonmedical Product Last 30 Days** |  |  |  |  |  |  |
| Yes | 78 (31.1%) | 50 (33.3%) | 28 (27.7%) | 15 (35.7%) | 10 (38.5%) | 5 (31.3%) |
| No | 169 (67.3%) | 97 (64.7%) | 72 (71.3%) | 27 (64.3%) | 16 (61.5%) | 11 (68.8%) |
| Don't Know or No Response | 4 (1.6%) | 3 (2.0%) | 1 (1.0%) | 0 (0%) | 0 (0%) | 0 (0%) |
| Boric Acid | 2 (0.8%) | 0 (0%) | 2 (2.0%) | 1 (2.4%) | 1 (3.8%) | 0 (0%) |
|  |  |  |  |  |  |  |
| **Reported Menstruation at Baseline** |  |  |  |  |  |  |
| Yes | 20 (8.0%) | 16 (10.7%) | 4 (4.0%) | 5 (11.9%) | 5 (19.2%) | 0 (0%) |
| No | 230 (91.6%) | 134 (89.3%) | 96 (95.0%) | 37 (88.1%) | 21 (80.8%) | 16 (100%) |
| No Response | 1 (0.4%) | 0 (0%) | 1 (1.0%) | 0 (0%) | 0 (0%) | 0 (0%) |
|  |  |  |  |  |  |  |
| **Current Spotting** |  |  |  |  |  |  |
| Yes | 32 (12.7%) | 20 (13.3%) | 12 (11.9%) | 9 (21.4%) | 6 (23.1%) | 3 (18.8%) |
| No | 218 (86.9%) | 130 (86.7%) | 88 (87.1%) | 33 (78.6%) | 20 (76.9%) | 13 (81.3%) |
| No Response | 1 (0.4%) | 0 (0%) | 1 (1.0%) | 0 (0%) | 0 (0%) | 0 (0%) |
|  |  |  |  |  |  |  |
| **Lifetime Sex Partners** |  |  |  |  |  |  |
| Male Sex Partners |  |  |  |  |  |  |
| 1-10 | 85 (33.9%) | 55 (36.7%) | 30 (29.7%) | 16 (38.1%) | 11 (42.3%) | 5 (31.25%) |
| 11-20 | 49 (19.5%) | 29 (19.3%) | 20 (19.8%) | 11 (26.2%) | 6 (23.1%) | 5 (31.25%) |
| >20 | 52 (20.7%) | 29 (19.3%) | 23 (22.8%) | 7 (16.7%) | 3 (11.5%) | 4 (25.0%) |
| No Response | 65 (25.9%) | 37 (24.7%) | 28 (27.7%) | 8 (19.0%) | 6 (23.1%) | 2 (12.5%) |
|  |  |  |  |  |  |  |
| Female Sex Partners |  |  |  |  |  |  |
| 0 | 133 (53.0%) | 82 (54.7%) | 51 (50.5%) | 18 (42.9%) | 12 (46.2%) | 6 (37.5%) |
| 1-10 | 86 (34.2%) | 55 (36.7%) | 31 (30.7%) | 19 (45.2%) | 12 (46.2%) | 7 (43.8%) |
| >10 | 10 (4.0%) | 7 (4.6%) | 3 (3.0%) | 1 (2.4%) | 0 (0%) | 1 (6.2%) |
| No Response | 22 (8.8%) | 6 (4.0%) | 16 (15.8%) | 4 (9.5%) | 2 (7.6%) | 2 (12.5%) |
|  |  |  |  |  |  |  |
| **New Sex Partner**  **Last 60 Days** |  |  |  |  |  |  |
| Yes | 76 (30.3%) | 44 (29.3%) | 32 (31.7%) | 13 (31.0%) | 7 (26.9%) | 6 (37.5%) |
| No | 172 (68.5%) | 105 (70.0%) | 67 (66.3%) | 28 (66.6%) | 19 (73.1%) | 9 (56.3%) |
| Don’t Know or Prefer Not to Answer | 3 (1.2%) | 1 (0.7%) | 2 (2.0%) | 1 (2.4%) | 0 (0%) | 1 (6.2%) |
| New Male Partner | 69 (27.5%) | 41 (27.3%) | 28 (27.7%) | 12 (28.6%) | 6 (23.1%) | 6 (37.5%) |
| New Female Partner | 13 (5.2%) | 4 (2.7%) | 9 (8.9%) | 2 (4.8%) | 1 (3.8%) | 1 (6.3%) |
