# Supplementary material for: Gardnerella Species and Their Association With Bacterial Vaginosis
Source: J Infect Dis. 2024 Jan 24;230(1):e171–81. doi: 10.1093/infdis/jiae026 (PMC11272073; doi:10.1093/infdis/jiae026)
Supplement: jiae026_Supplementary_Data [file jiae026_supplementary_data.zip › supp_table3.docx]

**Supplementary Table 3 |** Prevalence of *Gardnerella* species groups by BV diagnostic criteria

|  | Gardnerella 16S | G. vaginalis  cpn60 | G. piotii/ picketti  cpn60 | G. swidsinskii/ greenwoodii  cpn60 | G. leopoldii  cpn60 | Gardnerella species groups detected  (Mean ± SD) |
| --- | --- | --- | --- | --- | --- | --- |
| **Amsel Negative**  N=150 | 112  (74.7%) | 76  (50.7%) | 66  (44.0%) | 64  (42.7%) | 38  (25.3%) | 1.6 ±  1.4 |
| **Amsel Positive**  N=101 | 100  (99.0%) | 99  (98.0%) | 92  (91.1%) | 83  (82.2%) | 65  (64.4%) | 3.3 ±  0.7 |
| **Nugent Score 0-3**  N=115 | 80  (69.6%) | 47  (40.9%) | 35  (30.4%) | 38  (33.0%) | 23  (20.0%) | 1.2 ±  1.2 |
| **Nugent Score 4-6**  N=32 | 29  (90.6%) | 26  (81.3%) | 25  (78.1%) | 22  (68.8%) | 15  (46.9%) | 2.8 ±  1.2 |
| **Nugent Score 7-10**  N=103 | 103  (100%) | 102  (99.0%) | 98  (95.1%) | 87  (84.5%) | 65  (63.1%) | 3.4 ±  0.6 |
| **Asymptomatic BV/Intermediate Microbiota ^a^**  N=39 | 36  (92.3%) | 33  (84.6%) | 33  (84.6%) | 28  (71.8%) | 19  (48.7%) | 2.9 ±  1.1 |
| **Concordant BV Negative ^b^**  N=110 | 76  (69.1%) | 42  (38.2%) | 33  (30.0%) | 36  (32.7%) | 19  (17.3%) | 1.7 ±  1.0 |
| **Concordant BV Positive ^c^**  N=96 | 96  (100%) | 95  (99.0%) | 90  (93.8%) | 81  (84.4%) | 61  (63.5%) | 3.4 ±  0.6 |

^a^ Amsel negative and Nugent score 4-10.

^b^ Amsel negative and Nugent score 0-3.

^c^ Amsel positive and Nugent score 4-10.
