# Supplementary material for: Gardnerella Species and Their Association With Bacterial Vaginosis
Source: J Infect Dis. 2024 Jan 24;230(1):e171–81. doi: 10.1093/infdis/jiae026 (PMC11272073; doi:10.1093/infdis/jiae026)
Supplement: jiae026_Supplementary_Data [file jiae026_supplementary_data.zip › supp_table4a.docx]

**Supplementary Table 4A |** Performance characteristics of *Gardnerella* cpn60 qPCR assays for BV detection based on Nugent score (7-10 = BV; 0-6= Intermediate microbiota/No BV)

| **Bacteria Present Above Assay Threshold** | **Total (n=250)** | **Nugent**  **Score 7-10**  **(n=103)** | **Nugent Score 0-6 (n=147)** | **Sensitivity** | **Specificity** | **PPV** | **NPV** | **RR** | **95% CI** | **p-value** |
| --- | --- | --- | --- | --- | --- | --- | --- | --- | --- | --- |
| Gardnerella 16S rRNA | 212  (84.8%) | 103  (100%) | 109 (74.1%) | 100.0% | 25.9% | 48.6% | 100.0% | 36.9 | 2.3 –  ∞ | p<0.0001 |
| G. vaginalis | 175  (70.0%) | 102  (99.0%) | 73  (49.7%) | 99.0% | 50.3% | 58.3% | 98.7% | 43.7 | 8.1 - 247.9 | p<0.0001 |
| G. piotii/pickettii | 158  (63.2%) | 98  (95.1%) | 60  (40.8%) | 95.1% | 59.2% | 62.0% | 94.6% | 11.4 | 5.1 - 26.7 | p<0.0001 |
| G. swidsinskii/greenwoodii | 147  (58.8%) | 87  (84.5%) | 60  (40.8%) | 87.5% | 59.2% | 59.2% | 84.5% | 3.8 | 2.4 - 6.1 | p<0.0001 |
| G. leopoldii | 103  (41.2%) | 65  (63.1%) | 38  (25.9%) | 63.1% | 74.2% | 63.1% | 74.2% | 2.4 | 1.8 - 3.3 | p<0.0001 |
| 3 or more Gardnerella cpn60 species | 140  (56.0%) | 97  (94.2%) | 43  (29.3%) | 94.2% | 70.8% | 69.3% | 94.6% | 12.7 | 6.0 - 27.7 | p<0.0001 |
|  |  |  |  |  |  |  |  |  |  |  |
| **Quantity Detected Above Median Concentration of Positive Samples** | **Total (n=250)** | **Nugent Score 7-10 (n=103)** | **Nugent Score 0-6 (n=147)** | **Sensitivity** | **Specificity** | **PPV** | **NPV** | **RR** | **95% CI** | **p-value** |
| Gardnerella 16S rRNA  (>1.70e8 copies/swab) | 106  (42.2%) | 81  (78.6%) | 25  (17.0%) | 78.6% | 83.0% | 76.4% | 84.7% | 5.0 | 3.4 - 7.5 | p<0.0001 |
| G. vaginalis  (>2.45e7 copies/swab) | 87  (34.8%) | 66  (64.1%) | 21  (14.3%) | 64.1% | 85.7% | 75.9% | 77.3% | 3.3 | 2.5 - 4.6 | p<0.0001 |
| G. piotii/pickettii  (>3.48e6 copies/swab) | 79  (31.6%) | 51  (49.5%) | 28  (19.0%) | 49.5% | 81.0% | 64.6% | 69.6% | 2.1 | 1.6 - 2.8 | p<0.0001 |
| G. swidsinskii/greenwoodii  (>6.25e7 copies/swab) | 73  (29.2%) | 58  (56.3%) | 15  (10.2%) | 56.3% | 89.8% | 79.5% | 74.6% | 3.1 | 2.4 - 4.1 | p<0.0001 |
| G. leopoldii  (>4.28e7 copies/swab) | 51  (20.4%) | 34  (33.0%) | 17  (11.6%) | 33.0% | 88.4% | 66.7% | 65.3% | 1.9 | 1.4 - 2.5 | p<0.0001 |
