# Supplementary material for: Gardnerella Species and Their Association With Bacterial Vaginosis
Source: J Infect Dis. 2024 Jan 24;230(1):e171–81. doi: 10.1093/infdis/jiae026 (PMC11272073; doi:10.1093/infdis/jiae026)
Supplement: jiae026_Supplementary_Data [file jiae026_supplementary_data.zip › supp_table4b.docx]

**Supplementary Table 4B |** Performance characteristics of *Gardnerella* cpn60 qPCR assays for BV detection based on Nugent score (4-10 = Intermediate microbiota/BV; 0-3 = No BV)

| **Bacteria Present Above Assay Threshold** | **Total (n=250)** | **Nugent**  **Score 4-10**  **(n=135)** | **Nugent Score 0-3 (n=115)** | **Sensitivity** | **Specificity** | **PPV** | **NPV** | **RR** | **95% CI** | **p-value** |
| --- | --- | --- | --- | --- | --- | --- | --- | --- | --- | --- |
| Gardnerella 16S rRNA | 212  (84.8%) | 132  (97.8%) | 80  (69.6%) | 97.8% | 30.4% | 62.3% | 92.1% | 7.9 | 3.0-  23.0 | <0.0001 |
| G. vaginalis | 175  (70.0%) | 128  (94.8%) | 47  (40.9%) | 94.8% | 59.1% | 73.1% | 90.7% | 7.8 | 4.0-  16.0 | <0.0001 |
| G. piotii/pickettii | 158  (63.2%) | 123  (91.1%) | 35  (30.4%) | 91.1% | 69.6% | 77.9% | 87.0% | 6.0 | 3.6-  10.3 | <0.0001 |
| G. swidsinskii/greenwoodii | 147  (58.8%) | 109  (80.7%) | 38  (33.0%) | 80.7% | 67.0% | 74.2% | 74.8% | 2.9 | 2.1-  4.2 | <0.0001 |
| G. leopoldii | 103  (41.2%) | 80  (59.3%) | 23  (20.0%) | 59.3% | 79.1% | 77.7% | 61.3% | 2.0 | 1.6-  2.5 | <0.0001 |
| 3 or more Gardnerella cpn60 species | 140  (56.0%) | 119  (88.1%) | 21  (18.3%) | 88.2% | 81.7% | 85.0% | 85.5% | 5.8 | 3.8-  9.3 | <0.0001 |
|  |  |  |  |  |  |  |  |  |  |  |
| **Quantity Detected Above Median Concentration of Positive Samples** | **Total (n=250)** | **Nugent Score 4-10 (n=135)** | **Nugent Score 0-3 (n=115)** | **Sensitivity** | **Specificity** | **PPV** | **NPV** | **RR** | **95% CI** | **p-value** |
| Gardnerella 16S rRNA  (>1.70e8 copies/swab) | 106  (42.2%) | 99  (73.3%) | 7  (6.1%) | 73.3% | 93.3% | 93.4% | 73.1% | 3.5 | 2.7-  4.7 | <0.0001 |
| G. vaginalis  (>2.45e7 copies/swab) | 87  (34.8%) | 80  (59.3%) | 7  (6.1%) | 59.3% | 93.3% | 92.0% | 64.1% | 2.6 | 2.1-  3.2 | <0.0001 |
| G. piotii/pickettii  (>3.48e6 copies/swab) | 79  (31.6%) | 70  (51.9%) | 9  (7.8%) | 51.9% | 92.2% | 88.6% | 62.0% | 2.3 | 1.9-  2.9 | <0.0001 |
| G. swidsinskii/greenwoodii  (>6.25e7 copies/swab) | 73  (29.2%) | 69  (51.1%) | 4  (3.5%) | 51.1% | 96.5% | 94.5% | 62.7% | 2.5 | 2.1-  3.1 | <0.0001 |
| G. leopoldii  (>4.28e7 copies/swab) | 51  (20.4%) | 43  (31.9%) | 8  (7.0%) | 31.9% | 92.4% | 84.3% | 51.3% | 1.7 | 1.4-  2.1 | <0.0001 |
