# Supplementary material for: Gardnerella Species and Their Association With Bacterial Vaginosis
Source: J Infect Dis. 2024 Jan 24;230(1):e171–81. doi: 10.1093/infdis/jiae026 (PMC11272073; doi:10.1093/infdis/jiae026)
Supplement: jiae026_Supplementary_Data [file jiae026_supplementary_data.zip › supp_table5.docx]

**Supplementary Table 5|** Acquisition and loss of *Gardnerella* species groups in weekly longitudinal participants, n=42

|  | **G. vaginalis** | **G. piotii/ pickettii** | **G. swidsinskii/ greenwoodii** | **G. leopoldii** |
| --- | --- | --- | --- | --- |
| Participants with species  detection at baseline | 31/42  (73.8%) | 27/42  (64.3%) | 31/42  (73.8%) | 12/42  (28.6%) |
|  |  |  |  |  |
| Participants with species detection at baseline who lost species (any timepoint) | 11/31  (35.5%) | 12/27  (44.4%) | 9/31  (29.0%) | 9/12  (75.0%) |
| Participants with species detection at baseline and final time point (weeks 10-12) | 27/31  (87.1%) | 19/27  (70.4%) | 26/31  (83.9%) | 5/12  (41.7%) |
| Participants without species  detection at baseline | 11/42  (26.2%) | 15/42  (35.7%) | 11/42  (26.2%) | 30/42  (72.4%) |
| Participants without species detection at baseline who acquired species (any timepoint) | 2/11  (18.2%) | 3/15  (20%) | 5/11  (45.5%) | 8/30  (26.7%) |
| Participants without species detection at baseline and final time point (weeks 10-12) | 10/11  (90.9%) | 12/15  (80.0%) | 8/11  (72.7%) | 29/30  (96.7%) |
