# Supplementary material for: Gardnerella Species and Their Association With Bacterial Vaginosis
Source: J Infect Dis. 2024 Jan 24;230(1):e171–81. doi: 10.1093/infdis/jiae026 (PMC11272073; doi:10.1093/infdis/jiae026)
Supplement: jiae026_Supplementary_Data [file jiae026_supplementary_data.zip › supp_table6.docx]

**Supplementary Table 6 |** Summary of previous studies using PCR methods to link *Gardnerella* spp. to BV

| **Study** | **Study Type** | **Study Participants (n)** | **BV+ Participants (Method)** | ***Gardnerella* spp. Identification Method** | **BV Associated *Gardnerella* Clades/Species** |
| --- | --- | --- | --- | --- | --- |
| Balashov SV  et al. 2014 | Cross-sectional | 60 | 24 (Amsel and Nugent) | Clade specific qPCR^a^ | Clades 1 and 3 |
| Janulaitiene M et al. 2017 | Cross-sectional | 109 | 29 (Amsel and Nugent) | Clade specific qPCR^a^ | Clades 1 and 2 |
| Hilbert DW  et al. 2017 | Longitudinal | 149 | 123 (Amsel) | Clade specific qPCR^a^ | Clades 1, 2, 3, and 4 |
| Vodstrcil LA  et al. 2017 | Longitudinal | 52 | 7 (Nugent) | Clade specific qPCR^a^ | Clade 4 |
| Shipitsyna E  et al. 2019 | Cross-sectional | 299 | 79 (Nugent) | Clade specific qPCR^a^ | Clades 1, 2, and 4 |
| Plummer EL  et al. 2020 | Longitudinal | 101 | 48 (Nugent) | Clade specific qPCR^a^ | Clades 1, 2, and 3 |
| Hill JE  et al. 2019 | Cross-sectional | 417 | 64 (Nugent) | cpn60 sequencing^b^ | *G. vaginalis*, *G. piotii*, *G. swidsinskii* |
| Turner E  et al. 2021 | Longitudinal | 43 | 43 (Amsel) | cpn60 qPCR^c^ | genomospecies 7,  *G. swidsinskii/leopoldii* |

^a^Multiplex qPCR targeting clade specific markers for *Gardnerella* clades 1-4 developed by Balashov SV et al.

^b^High throughput sequencing of cpn60 gene measuring relative abundance of 13 *Gardnerella* genomospecies.

^c^qPCR assays targeting cpn60 gene of *G. vaginalis* and genomospecies 2, *G. piotii* and genomospecies 3 (*G. pickettii*), *G. swidsinskii* and G. *leopoldii*, *Gardnerella* genomospecies 7, and *Gardnerella* genomospecies 8-10.
